# Painless Thyroiditis by Withania somnifera (Ashwagandha)

**DOI:** 10.7759/cureus.55352

**Published:** 2024-03-01

**Authors:** Maho Hayashi, Hina Hamada, Shin-ichiro Azuma, Koji Hayashi

**Affiliations:** 1 Department of Internal Medicine, Fukui General Hospital, Fukui, JPN; 2 Department of Rehabilitation Medicine, Fukui General Hospital, Fukui, JPN

**Keywords:** ashwagandha (withania somnifera), thyrotoxicosis, subacute lymphocytic thyroiditis, drug-induced thyrotoxicosis, painless thyroiditis

## Abstract

The *Withania somnifera*, also called Ashwagandha, is available everywhere in the world. We present a rare case of thyrotoxicosis following Ashwagandha administration, specifically painless thyroiditis (PT) in this report. The patient was a 47-year-old previously healthy Japanese man, who started taking Ashwagandha two months before his first visit to our hospital. He visited our hospital for typical thyrotoxicosis symptoms like a sense of fatigue, fever at night, and weight loss followed by diarrhea and headache. Blood tests disclosed thyrotoxicosis. Thyroid ultrasonography showed internal echo heterogeneity and no increase in blood flow. Thyroid scintigraphy revealed a deficiency in thyroid uptake. Based on these findings, he was diagnosed as PT. After stopping the administration of Ashwagandha, both his symptoms and serum thyroid markers were improved. This report may spark important debate about whether ashwagandha is safe among healthy people, especially in thyroid toxicity.

## Introduction

The *Withania somnifera*, known commonly as ashwagandha or winter cherry, is an evergreen shrub in the Solanaceae or nightshade family that grows in India [1,2]. This herb has been used in traditional oral herbal therapy in India [1]. Ashwagandha is still used for conditions such as arthritis, asthma, goiter, and ulcers, as well as anxiety, insomnia, and neurological disorders [1], by the effect of adaptogenic, anti-stress, and anti-inflammatory properties related to its plant [2]. A lot of products of Ashwagandha can be available in many countries including the US and East Asia [1]. According to the National Institutes of Health Office of Dietary Supplements in the US, more than 1,300 products that contain Ashwagandha are currently available, and its use is becoming increasingly common [1]. Other hand, the adverse effects of Ashwagandha are less recognized.

Thyroid hormone is controlling metabolism, growth, and many other bodily functions [3]. Thyrotoxicosis is the clinical manifestation of excess thyroid hormone action at the tissue level due to inappropriately high circulating thyroid hormone concentrations [4]. One of the causes of thyrotoxicosis is thyroiditis. Thyroiditis is a group of inflammatory thyroid disorders [5]. Thyroiditis includes chronic lymphocytic thyroiditis (also referred to as Hashimoto's thyroiditis), subacute granulomatous thyroiditis, subacute lymphocytic thyroiditis (SLT), and acute (suppurative) thyroiditis [5]. To differentiate between these diseases, blood tests, thyroid function evaluation (increased or decreased thyroid hormone), thyroid echo test, nuclear medicine examination, palpation, etc., are used. Of these conditions, SLT is subdivided into two groups, postpartum thyroiditis and sporadic painless thyroiditis (PT) [5]. SLT starts with an initial hyperthyroid phase, followed by subsequent hypothyroidism and, finally, a return to the euthyroid state [5].

Recently, Ashwagandha's effects on decreased thyroid function have attracted attention. A double-blind, randomized placebo-controlled trial for patients with subclinical hypothyroidism shows normalized serum thyroid hormone [6]. However, reports of hyperthyroidism, especially thyrotoxicosis by Ashwagandha among healthy populations are extremely rare. Herein, we report a rare case of Ashwagandha-induced PT in a healthy man. 

## Case presentation

A 47-year-old Japanese man developed insomnia and started taking Ashwagandha extract two months before his first visit to our hospital. He was compliant with the Ashwagandha dosage. He was a bodybuilder and was previously healthy. Ten days before the first visit, he suffered from a sense of fatigue and fever at night. In addition, he complained of weight loss of 4 kg in two weeks and visited our hospital. Vital signs were unremarkable except slight fever (37.1 degrees Celsius). Thyroid palpation revealed no thyroid enlargement or tenderness. No hand tremor was noted. Blood tests disclosed elevated white blood cells (11,800/μL) and CRP (1.93 mg/dL). Biochemical analysis related thyroid function showed elevated free triiodothyronine (FT3) and free thyroxine (FT4) and decreased thyroid-stimulating hormone (TSH), which disclosed thyrotoxicosis, but near normal results for TSH receptor antibodies (TRAb) and thyroid-stimulating antibodies (TSAb), which was unlikely to have Basedow’s disease (Table 1). In addition, both anti-thyroid peroxidase antibodies (TPOAb) and anti-thyroglobulin antibodies (TgAb) were negative, which was unlikely to have subclinical Hashimoto’s thyroiditis (Table 1).

**Table 1:** The result of blood tests of biochemical analysis related thyroid gland in first visit.

Inspection items	Result	Reference range
Free triiodothyronine (FT3)	6.1 pg/mL	(2.3–4.0)
Free thyroxine (FT4)	2.8 ng/dL	(0.9–1.7)
Thyroid stimulating hormone (TSH)	0.001 μIU/mL	(0.35–4.94)
Thyroglobulin (Tg)	464 ng/dL	(<46)
Thyroid stimulating hormone receptor antibodies (TRAb)	1.1 IU/mL	(<1)
Thyroid stimulating antibodies (TSAb)	92%	(<120)
Anti-thyroglobulin antibodies (TgAb)	13.6 IU/mL	(<28)
Anti-thyroid peroxidase antibodies (TPOAb)	<9.0 IU/mL	(<16)

**Figure 1:**
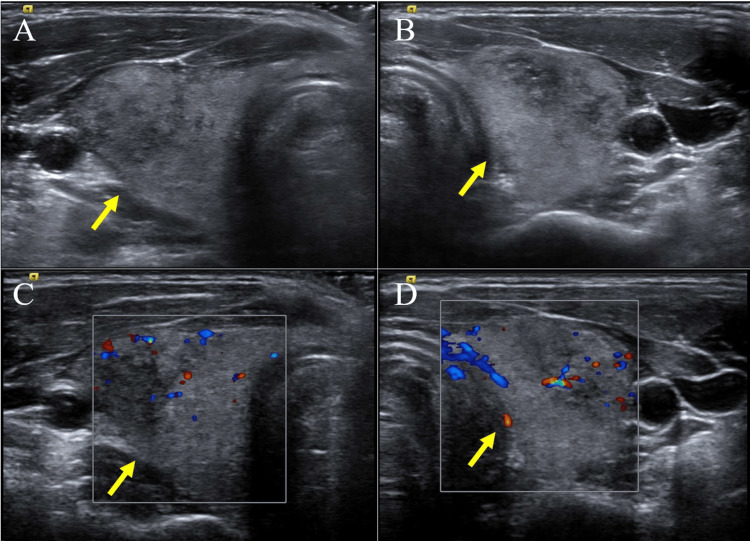
The result of thyroid ultrasonography. Thyroid ultrasonography (US) showing slightly enlargement of thyroid gland and internal heterogeneity (A: right lobe, B: left lobe). Color flow Doppler enhancement of the US images showing no increase in blood flow (C: right lobe, D: left lobe).

**Figure 2:**
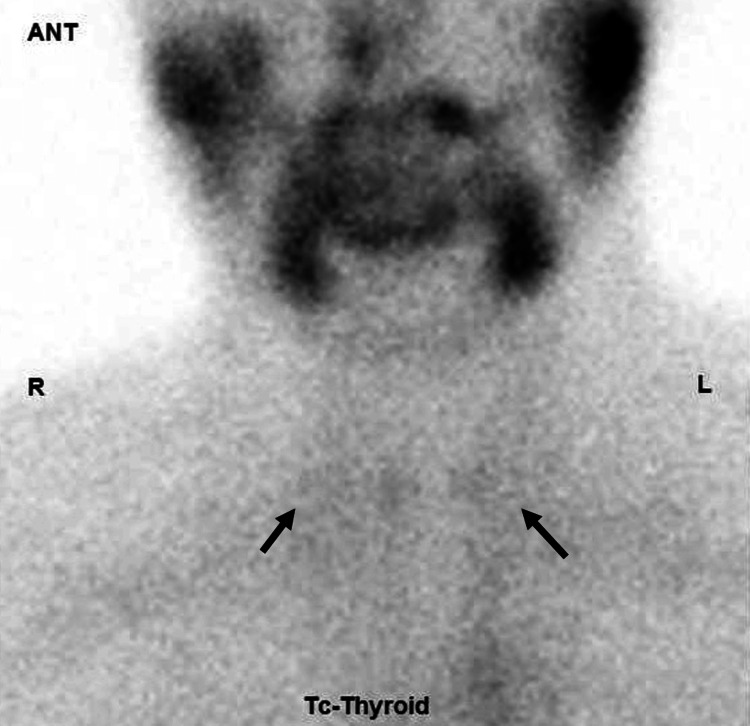
The result of thyroid 99mTcO4 scintigraphy. Thyroid ^99m^TcO_4_ scintigraphy showing almost no uptake in thyroid gland (arrowheads).

Thyroid ultrasonography showed internal echo heterogeneity, and no increase in blood flow (Figures 1A-1D). Additionally, thyroid 99mTcO4 scintigraphy revealed a deficiency in thyroid uptake (Figure 2).

**Figure 3:**
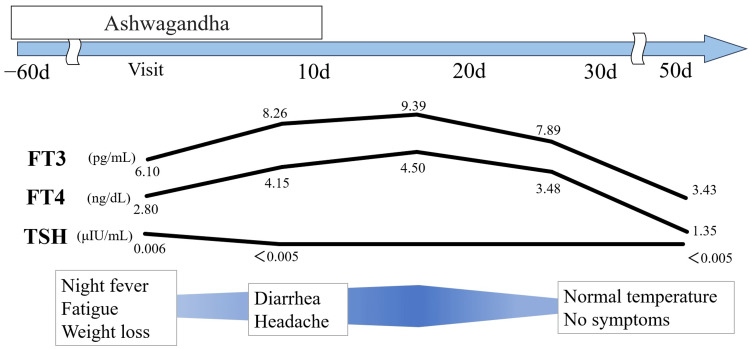
Schema of clinical course in our case. The initial visit to our hospital was defined as day 0. Symptoms were developed after taking Ashwagandha and recovered after stopping it. The levels of thyroid marker showing in this schema including free triiodothyronine (FT3), free thyroxine (FT4) and thyroid stimulating hormone (TSH). After stopping administration of Ashwagandha, FT3 and FT4 were decreased within reference range.

On the basis of typical symptoms of thyrotoxicosis (including a sense of fatigue, fever, weight loss, diarrhea, and headache) and Physiological/radiological test results, we diagnosed him as PT, which is a subgroup of SLT. The subsequent progress is shown in Figure 3. The patient returned to our hospital seven days after the initial visit. As we could not avail the result of thyroid function and did not notice the relationship between thyrotoxicosis and Ashwagandha at the first visit, he did not stop taking Ashwagandha. His symptoms were not improved, but exacerbated; diarrhea and headache were developed in addition to the original symptoms. Blood markers related to thyroid function were also worsened including FT3 and FT4. We warned him that he had thyrotoxicosis, a side effect of Ashwagandha. He stopped taking it 10 days after the initial visit. He revisited our hospital on 14 days and his clinical symptoms were not improved. His blood tests revealed elevated thyroid markers. However, after this day, both clinical symptoms and blood thyroid markers were improved. After 50 days, his symptoms were completely recovered and his thyroid status became euthyroid. This clinical course was consistent with thyrotoxicosis induced by medications. In addition, blood, physiological, and radiological examinations supported PT. He did not visit our hospital for subsequent follow-up.

## Discussion

We describe a rare case of Ashwagandha-induced PT in a healthy man. He had symptoms related to thyrotoxicosis after taking Ashwagandha and his symptoms were recovered after discontinuing it. Additionally, changes in serum thyroid markers were dependent on the timing of Ashwagandha administration. PT is diagnosed on the basis of an increased thyroglobulin level, a low radioactive iodine uptake, an increased FT4 level, and a suppressed TSH level [7]. In other criteria, the characteristics of PT are no neck pain, hyperthyroidism, and decreased radioactive iodine uptake [5]. Based on these criteria, our patient was diagnosed as PT. However, although both TPOAb and TgAb were negative in our case, it is reported that TgAb is present in 50% to 80% of SLT including PT, while antiTPOAb are present in nearly all patients [5]. Therefore, our case may be more accurately called drug-induced destructive thyroiditis or drug-induced thyrotoxicosis.

Although the mechanism that Ashwagandha causes PT remains unclear, some studies about Ashwagandha and thyroid function have been reported. In the mice model, the administration of Ashwagandha root extract for 20 days increases serum triiodothyronine (T3) and thyroxine (T4) [8]. In addition, the subsequent study revealed that the administration of Ashwagandha to mice for 20 days caused a significant increase in the level of serum T4 reaching approximately 111% compared to only an 18% increase in serum T3 [9]. Authors of these studies speculated that the etiology of elevated thyroid hormone is caused by stimulation to the thyroid gland by Ashwagandha, and Ashwagandha root extract might act both as a prothyroidic agent and as an antiperoxidative agent [9]. In addition, it has been reported that the effect of Ashwagandha on the cortex and hippocampus in rats with drug-induced hypothyroidism is evaluated [10]. In this report, rats treated with ashwagandha had significantly higher serum T3 and T4 levels than those not treated. Although the authors of this study point out that ashwagandha reduces the nervous system and oxidative stress, it is possible that the effect is due to the effect of the thyroid hormone itself, via increasing serum thyroid hormone increased by Ashwagandha.

In humans, some studies have reported about Ashwagandha and thyroid function. In healthy volunteers, Ashwagandha causes elevated FT3 and FT4 without any adverse effects [11]. Additionally, a placebo-controlled study of Ashwagandha in patients with bipolar disorder revealed elevated FT4, but no statistically significant difference [12]. Moreover, a double-blind, randomized placebo-controlled trial of Ashwagandha for hypothyroidism patients disclosed that Ashwagandha improved serum TSH, T3, and T4 levels significantly compared to placebo [6]. Among the above studies in humans, there were no significant side effects. Furthermore, the detailed mechanism affecting thyroid function remained unclear.

Regarding the unfavorable effect of Ashwagandha on thyroid function in humans, as far as we know, there are three reports of thyrotoxicosis [13-15]. Of three reports, thyrotoxicosis is noted in two previously healthy people and one hypothyroidism patient [13-15]. One healthy case had an enlargement of the thyroid gland and pain on thyroid palpation or by swallowing [14]. The presence of neck pain suggests subacute granulomatous thyroiditis or microbial inflammatory thyroiditis [5]. Therefore, this case is quite different from the characteristics of our case. The other healthy case developed thyrotoxicosis after increasing the dose of Ashwagandha [15]. Because this article is not English literature, we could not read it in detail. In one hypothyroidism case, there was no evidence of thyroid enlargement, thyroid nodules, or tenderness of the thyroid gland [13]. This case was positive for thyroid microsomal antibody, representing Hashimoto’s thyroiditis. No one out of three had nuclear medicine tests performed. Therefore, none out of the three previous cases are diagnosed as PT like our case.

Our case may spark an interesting debate about whether Ashwagandha is safe among healthy people, especially in thyroid function. Because PT can sometimes cause thyroid storm [16] or lead to permanent hypothyroidism in the chronic phase [5], administration of Ashwagandha for the purpose of improving thyroid function requires careful judgment, especially when symptoms of thyrotoxicosis appear. Further studies are needed to reveal the underlying mechanism of Ashwagandha-induced PT.

## Conclusions

We presented an interesting case with PT caused by Ashwagandha. Whereas detailed mechanisms of Ashwagandha and PT remain unknown, previous reports supported thyrotoxicosis induced by Ashwagandha. Nowadays, Ashwagandha is available in many streets around the world. This paper may spark an interesting debate about its safety.
